# Neurodynamics in the Sensorimotor Loop: Representing Behavior Relevant External Situations

**DOI:** 10.3389/fnbot.2017.00005

**Published:** 2017-02-03

**Authors:** Frank Pasemann

**Affiliations:** Institute of Cognitive Science, University of OsnabrückOsnabrück, Germany

**Keywords:** neurodynamics, behavior control, sensorimotor loop, mathematical concepts, neural representations

## Abstract

In the context of the dynamical system approach to cognition and supposing that brains or brain-like systems controlling the behavior of autonomous systems are permanently driven by their sensor signals, the paper approaches the question of neurodynamics in the sensorimotor loop in a purely formal way. This is carefully done by addressing the problem in three steps, using the time-discrete dynamics of standard neural networks and a fiber space representation for better clearness. Furthermore, concepts like meta-transients, parametric stability and dynamical forms are introduced, where meta-transients describe the effect of realistic sensor inputs, parametric stability refers to a class of sensor inputs all generating the “same type” of dynamic behavior, and a dynamical form comprises the corresponding class of parametrized dynamical systems. It is argued that dynamical forms are the essential internal representatives of behavior relevant external situations. Consequently, it is suggested that dynamical forms are the basis for a memory of these situations. Finally, based on the observation that not all brain process have a direct effect on the motor activity, a natural splitting of neurodynamics into vertical (internal) and horizontal (effective) parts is introduced.

## 1. Introduction

From a neurocybernetics perspective the dynamical systems approach to embodied cognition can be traced back to the work of Ashby (Ashby, 1960) and von Foerster (Von Foerster, 1960). The assumption is that a living organism, in order to survive, must be able to develop internally some stable “entities” (von Foerster) which refer to or classify objects and situations in the physical world. These “entities” are the result of cognitive and sensorimotor processes developing through continuous interactions of an individual with its specific environment. On the other hand, cognitive and sensorimotor processes, relevant for the behavior of the individual, depend on the formation of these stable structures; i.e., they are complementary in the sense that one defines or implies the other. The assumption was, that an organism must be able to relate *discrete* internal structures to relevant aspects of its own interaction with its environment.

Although, the underlying processes are continuous these internal “entities” have to be discrete because the referenced objects or situations are discrete features of the environment. They also have to be “stable” in a certain time domain. On the other hand, due to changing sensorimotor or cognitive processes, they have to get “unstable” in the sense that different references have to be built up; i.e., new “stability domains” have to be visited or formed.

To pursue the dynamical systems approach to embodied cognition in this spirit, this paper will consider an individual as an autonomous system called an *animat*. An animat Dean (1998) and Guillot and Meyer (2001) is a simulated or physical robot equipped with sensors and actuators, and a neural network for behavior control. The neural controllers then have to operate in the so called *sensorimotor* loop, getting inputs from sensory signals and generating motor signals, which in turn will lead to new sensor inputs. The essential role of these closed loop processes for living or live-like systems has been discussed over several decades now from various points of view (Bishop, 1960; Beer, 1995; Di Paolo, 2003; Philipona et al., 2004; Hülse et al., 2007; Zahedi et al., 2010; Sándor et al., 2015). Here we use a purely formal approach and carefully analyze the dynamical description by making successive approximations to these processes.

Neurocontrollers, mimicking their biological counterparts, are considered as recurrent neural networks which in general allow for dynamical properties. That is, for fixed synaptic weights, bias terms and inputs such a network can be described as a dynamical system. Then, assuming that a neurocontroller is driven by slow sensor inputs, it will be properly described as a parametrized family of dynamical systems, where sensor inputs (and proprioceptive signals as well) are considered in a first approximation as parameters of such a family. Furthermore, for every parameter value the corresponding dynamical system may have a manifold of different attractors. The postulated internal “entities” then will be identified with the basins of attraction of *parametrically stable* neurodynamical systems. The interaction with the environment then may change the references to situations in the external world by changing parameter values given, for instance, by the sensor signals. This process of changing references will be described by so-called *bifurcations*.

For theoretical reasons, parameters are assumed to change so slowly that the system can approach its asymptotic states. This is often not the case for realistic sensor inputs. So, in a second step we will introduce sequences of neural states called *meta-transients* as for instance in Negrello and Pasemann (2008) Negrello (2011), and Toutounji and Pipa (2014).

In general these meta-transients can not be given an interpretation as trajectories of a dynamical system, mainly because the inducing sequence of sensor signals is not a trajectory of a dynamical system on sensor space. Instead, because of the closed loop, it is superposition of movements in the environment and the result of motor actions. The case where one has access to controlling parameters has often been discussed in geometric control theory (Gardner, 1983; Sussmann, 1983; Respondek, 1996; Kloeden et al., 2013). There then one can generalize the concept of attractors and the like. Although we do not find this approach applicable for the dynamics in the sensorimotor loop we will work with a comparable view.

Finally, these meta-transients have to be mapped to motor neurons, inducing then actions of the animats body; i.e., its behavior. Due to this projection not all elements of the neural system will be involved directly in the generation of motor signals. This leads naturally to a fiber structure over the motor space allowing to introduce the concepts of vertical or *internal* neurodynamics, having no direct effect on behavior, and a horizontal or *effective* neurodynamics, the projection of which generates the movements of the animat.

To clarify concepts, the paper will address the discrete-time neurodynamics of networks composed of standard sigmoidal neurons of additive type. Using this simplifying setup, it is assumed that the aspects described in the following are transferable also to neural systems employing more biologically plausible or other types of neurons. The basic concern here is to specify the role of, for example, attractors, basins of attraction, transients, bifurcations and stability properties in the context of systems acting in a sensorimotor loop.

Approaching the description of neurodynamics in the sensorimotor loop in three steps, we will first define the type of neurodynamics studied in this paper (Section 2), exemplifying it by some well known results. Assuming that sensor inputs are slow when compared to the activity dynamics of the neural system, we argue in Section 3 that neural systems in the sensorimotor loop are effectively described by parametrized families of dynamical systems, were parameters correspond to the sensor inputs. Other parameters, not considered here, are, for instance, signals coming from proprioceptors and the synaptic weights of the network, the change of which usually is associated with learning. Referring to the more realistic situations, meta-transients are introduction in Section 4. Finally, Section 5 discusses the generation of motor signals resulting from a projection of attractor transients or meta-transients, respectively, to the motor space; this then allows to differentiate between so called effective and internal neurodynamics. Finally the sensorimotor loop is closed through the environment by a formal mapping from motor space *M* to sensor space *S*. The paper concludes with a discussion of the possible role the introduced concepts can play for understanding neural representations of behavior relevant situations in the external world and, correspondingly, for a notion of memory which is not based on specific attractors like, for instance, fixed point attractors in Hopfield networks.

## 2. Neurodynamics

Besides the body of an animat, three different parts of it will be discerned: The “brain” considered as a recurrent neural network *N* with *n* neurons. Its sensor neurons will prescribe the sensor space *S*, and the output neurons will define the motor space *M*. Sensor space *S* and motor space *M* are the interfaces of the “brain” *N* to the physical world. Assuming strictly the point of view of an animat, the world for an animat is what happens on its sensor surface. We describe these parts more concrete as follows.

A state *a*(*t*) ∈ *A* ⊂ ℝ^*n*^ of the neural system *N* at time *t* is characterized by the activation of all its *n* neurons. Correspondingly, the state space *A* is called the *activation space* or phase space of *N*. It is a manifold of dimension *dim* (*A*) = *n*. Neural states may be represented in an equivalent way by the outputs *o*(*t*) of the *n* neurons, and we call the corresponding state space of the network *N* its output space A^*^.

The *sensor space S* consists of all possible sensor inputs, i.e., a sensor state *s*(*t*) ∈ *S* at time *t* consists of all sensor values at time *t*. The sensor space is assumed to be a bounded manifold *S* of dimension dim *S* = *m*, where *m* denotes the number of distinct sensor elements. *S* may be subdivided into modality spaces corresponding, for example, to visual, acoustic, or haptic inputs.

A motor state *m*(*t*) ∈ *M* at time *t* is given by the activation of the motor neurons at time *t* driving the various actuators of the animat. Thus, the *motor space* of the animat is an open bounded manifold *M* of dimension dim *M* = *k*, the number of all its motor neurons. The motor space *M* may be segmented into different domains responsible e.g., for head movement, eye movement, driving wheels, arms, and so forth. Special domains may be related to corresponding domains in sensor space: Fixed infrared sensors may be, for instance, related only to the wheels domain; but with pan-tilt-camera vision is related to wheels, and pan-tilt-motors, et cetera.

### 2.1. Discrete-time neurodynamics

For a general introduction into the theory of dynamical system see for example (Abraham and Shaw, 1992; Hirsch et al., 2012; Strogatz, 2014). Here, in a first approximation we will understand the neural system *N* as a discrete-time dynamical system (Kloeden et al., 2013); i.e., on its activation space *A* there exists a differentiable map ϕ : ℤ × *A* → *A*, called the *flow*, with the following properties:

- ϕ(0, *a*_0_) = *a*_0_, for all *a*_0_ ∈ *A*.
- ϕ(*s* + *t, a*_0_) = ϕ(*s*, ϕ(*t, a*_0_)), for all *s, t* ∈ ℤ and *a*_0_ ∈ *A*,

were ℤ denotes the set of nonnegative integers. In the following we consider a neural network *N* with activation space *A* ⊂ ℝ^*n*^, writing it as *N*(*A*), which is composed of *n* standard additive neurons with sigmoid transfer function τ: = tanh. The flow of this system is then generated by a diffeomorphism *f* : *A* → *A* given in component form by

(1) $$\begin{array}{l} a_{i}(t+1):=\theta_{i}+\sum_{j=1}^{n}w_{ij}\tau \left(a_{j}\left(t\right)\right),\;i=1,\ldots ,n, \end{array}$$

where θ_*i*_ represents a constant bias term of neuron *i*, *w*_*ij*_ the synaptic strength or weight from neuron *j* to neuron *i*, and τ denotes the transfer function. Thus, the output of neuron *i* is given by *o*_*i*_: = τ(*a*_*i*_), and for the output space we have A^*^ ⊂ (−1, 1)^*n*^.

The neural system *N*(*A*), considered as a dynamical system, will be denoted by (*A, f*). In this section terms like the bias terms θ_*i*_ and synaptic weights *w*_*ij*_ are assumed to be constant. This means that we consider an isolated system; i.e., there is no neural plasticity involved, and sensor inputs are not considered.

Furthermore, we endow the vector space *A* with an Euclidean metric *d*_τ_ induced by the transfer function τ; i.e.,

$$\begin{array}{l} d_{\tau }\left(a,a\prime \right):=d\left(\tau \left(a\right),\tau \left(a\prime \right)\right)=\sqrt{\overset{n}{\underset{i=1}{\sum }}{\left(\tau \left(a_{i}\right)-\tau \left(a_{i}^{\prime }\right)\right)}^{2}}. \end{array}$$

Due to the saturation domains of the sigmoid τ the distance of activity states corresponding to very high (positive or negative) activations is very small.

The flow on the state space *A* is then defined by

$$\begin{array}{l} \phi \left(t,a_{0}\right):=a\left(t\right)=f^{t}\left(a_{0}\right)=\underset{t\;times}{\underset{︸}{f◦f◦\ldots ◦f◦f}}\left(a_{0}\right), \end{array}$$

where *a*_0_ ∈ *A* is called the *initial state*. The flow ϕ satisfies the group property; i.e., with initial condition ϕ(0, *a*_0_) = *a*_0_ one has

$$\begin{array}{l} \phi \left(n,\phi \left(m,a_{=}\right)\right)=f^{n}◦f^{m}\left(a_{0}\right)=f^{n+m}\left(a_{0}\right)=\phi \left(n+m,a_{0}\right). \end{array}$$

**Figure 1:**
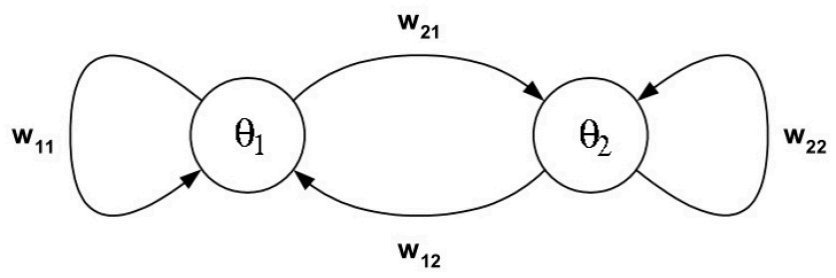
**A 2-neuron network**.

**Example 1:** The dynamics of 2-neuron networks have been analyzed extensively, in the continuous-time case as well as the discrete-time case, because already these simple systems, under certain conditions, can show all possible dynamical features: They can exhibit fixed point attractors as well as periodic, quasiperiodic and chaotic attractors, and even show co-existing attractors for one and the same condition (Wilson and Cowan, 1972; Marcus and Westervelt, 1989; Wang, 1991; Beer, 1995). Here we recall some of the results, which can be found for example in Pasemann (2002), to demonstrate basic properties of recurrent neural networks for this most simple case. So, let (*A, f*) denote the two-dimensional system given by two neurons (compare Figure 1) satisfying the equations

(2) $$\begin{array}{lll} a_{1}\left(t+1\right) & := & \theta_{1}+w_{11}\tau \left(a_{1}\left(t\right)\right)+w_{12}\tau \left(a_{2}\left(t\right)\right),\\ a_{2}\left(t+1\right) & := & \theta_{2}+w_{21}\tau \left(a_{1}\left(t\right)\right)+w_{22}\tau \left(a_{2}\left(t\right)\right). \end{array}$$

As a bounded dissipative dynamical systems, the time development of neural states can be characterized by attractors and transients. We first recall some basic definitions.

A time-sequence of states

(3) $$\begin{array}{l} O\left(a_{0}\right):=\left\{a_{0},a\left(1\right),\ldots ,a\left(t\right),\ldots \right\},\;a_{0}\in A, \end{array}$$

is called an *orbit* or a *trajectory* of the system starting from *a*_0_ ∈ *A*. An orbit *O*(*a*_0_) is called *periodic* of period *p* ≥ 1 if *a*(*p*) = *a*_0_, and *p* is the smallest integer such that this equation holds. For *p* = 1 the orbit is called a stationary state or a *fixed point* of the system. A *p-periodic point* is a state on a *p*-periodic orbit *O*(*a*_0_) = {*a*_0_, *a*(1), …, *a*(*p*)}. It corresponds to a fixed point of the *p*-th iterate *f*^*p*^ of the map *f*:

$$\begin{array}{l} f^{p}\left(a\right):=\underset{p-times}{\underset{︸}{f◦f◦\cdots ◦f}}\left(a\right)=a,\;a\in A. \end{array}$$

Let *U* ⊂ *A* denote a subset which is *invariant* under the action of *f*; i.e. *f*(*U*) = *U*. A closed and bounded set Γ ⊂ *U* is called an *attractor* of the dynamical system (*A, f*), if *f*(Γ) = Γ and there exists an ε > 0 such that

$$\begin{array}{l} d\left(a_{0},\Gamma \right)\leq \varepsilon ,a_{0}\in U,\text{ implies that }d\left(a\left(t\right),\Gamma \right)\to 0\text{ as }t\to \infty . \end{array}$$

**Figure 2:**
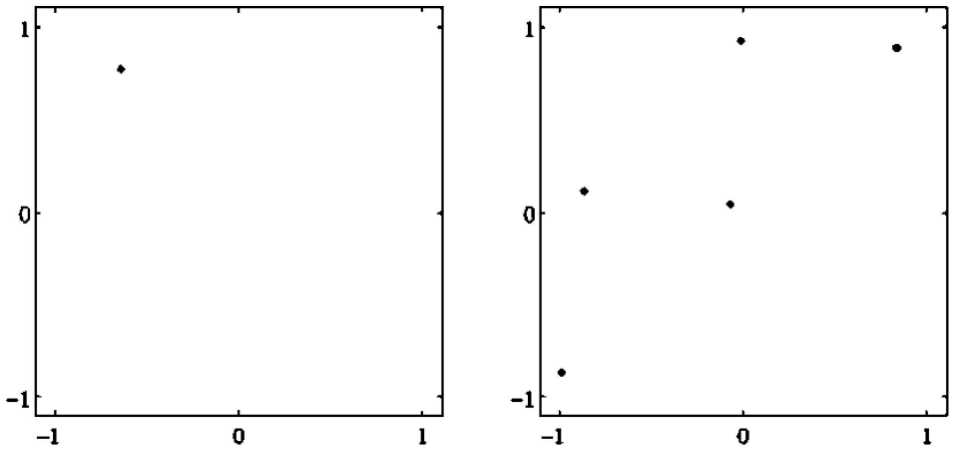
**Examples of attractors in (***o***_**1**_, ***o***_**2**_)-output space for a two neuron system (2). (Left)** A fixed point attractor. **(Right)** A period-5 attractor. (Parameters are given in Table A1 in the Appendix referring to networks sys1 and sys2.)

**Figure 3:**
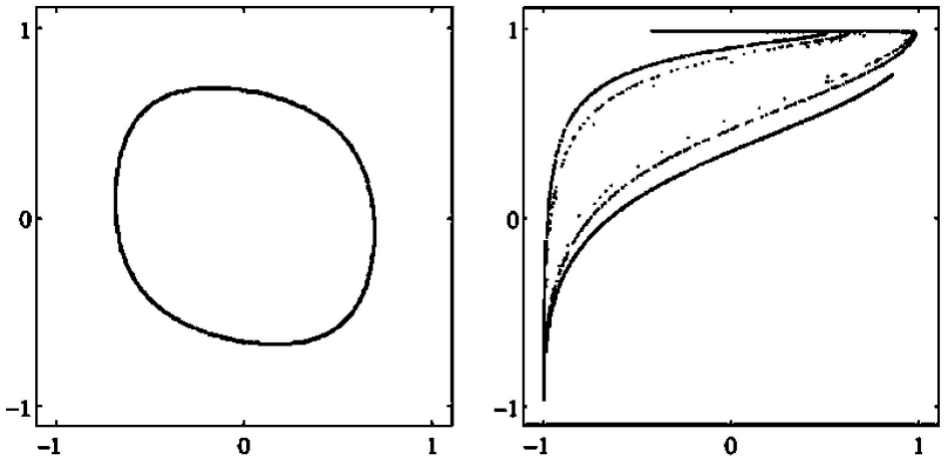
**Examples of attractors in (***o***_**1**_, ***o***_**2**_)-output space for a two neuron system (2). (Left)** A quasiperiodic attractor. **(Right)** A chaotic attractor. (Parameters are given in Table A1 in the Appendix referring to networks sys3 and sys4.)

There are different types of attractors: Fixed points, periodic orbits (a finite set of periodic points) as in Figure 2, *quasiperiodic orbits* represented by a dense set of points on a closed line, and so called *chaotic attractors* which are characterized, for instance, as a fractal set in *A* (compare also Figure 3 and Abraham and Shaw, 1992; Hirsch et al., 2012; Strogatz, 2014). If Γ is the only attractor of a system (*A, f*), then it is called a *global* attractor.

The *basin of attraction B*(Γ) of an attractor Γ is the set of all initial conditions *a*_0_ ∈ *A* such that *d*(*a*(*t*), Γ) → 0 as *t* → ∞. Thus, the basin of attraction of Γ is considered as the set of all orbits attracted by Γ. A *transient O*(Γ) of a system (*A, f*) is an orbit in the basin of an attractor Γ.

**Figure 4:**
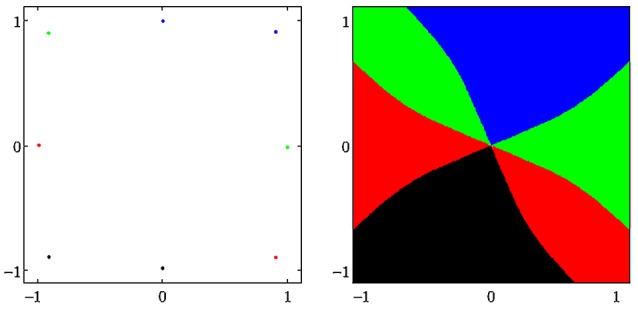
**(Left)** Four co-existing period-2 attractors in (*o*_1_, *o*_2_)-output space. **(Right)** Their basins of attraction (for parameters see Table A1, network sys5).

**Figure 5:**
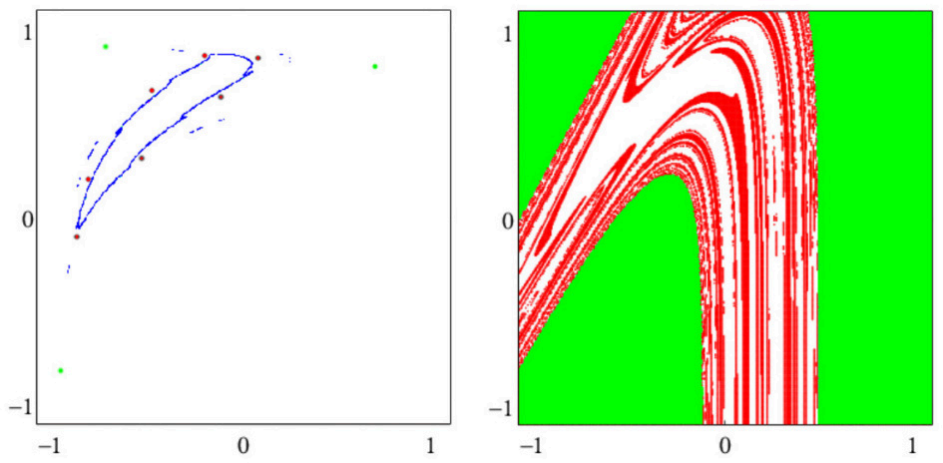
**(Left)** A period-3 attractor (green) and a period-7 attractor (red) in (*o*_1_, *o*_2_)-output space, co-existing with two chaotic attractors, one cyclic with period 14. **(Right)** The corresponding basins of attraction; the two basins of the chaotic attractors are white. The system is given as sys6 in Table A1.

A dynamical system (*A, f*) can have more than one attractor. Then we say that the system has several *co-existing* attractors. For instance, in Figure 4 four co-existing period-2 attractors and their basins with regular boundaries are shown. Figure 5 displays several co-existing attractors separated by fractal basin boundaries.

Often one uses the metaphor “landscape” to describe a dynamical system (*A, f*) qualitatively. This refers exactly to what we defined as the flow of the dynamical system (*A, f*). One can think about water running downhill into a sink when referring to transients approaching an attractor. Basin boundaries then correspond to water partings. An *attractor-landscape*, denoted by [*A*], then visualizes the different types of attractors present in the system together with their basins of attraction and basin boundaries as shown in the figures above.

Two different dynamical systems can have similar landscapes in the sense that there is the same number and type of attractors involved; but attractors, as well as the corresponding basin boundaries, may be deformed with respect to each other. If one can map the attractor-landscape of one system onto the attractor-landscape of the other system such that orbits are mapped one-to-one onto each other by preserving the time direction, then the qualitative behavior of such systems is comparable. This situation is formalized by the following

Definition 1. *Two discrete-time dynamical systems* (*A, f*) *and* (*B, g*) *are said to be* topologically conjugate, *if there exists a homeomorphism* ψ : *A* → *B, such that f* ◦ ψ = ψ ◦ *g, i.e., such that the following diagram commutes:*

$$\begin{array}{c} f\\ \psi \begin{array}{c} \begin{array}{cc} A & \begin{array}{cc} \to & A \end{array} \end{array}\\ \begin{array}{cc} ↓ & \begin{array}{cc} \; & ↓ \end{array} \end{array}\\ \begin{array}{ccc} B & \to & B \end{array} \end{array}\psi \\ g \end{array}$$

## 3. Parametrized families of dynamical systems

In the last section the bias terms θ_*i*_ and synaptic weights *w*_*ij*_, *i, j* = 1, …, *n*, were held constant, and one can consider them as parameters of the neural system (*A, f*). For different bias terms or synaptic weight one gets different dynamical systems. Thus, we introduce a *parameter space Q* ⊂ ℝ^*q*^ for a neural system (*A, f*) as a *q*-dimensional Euclidean manifold (*Q, h*) with metric *h*. A parameter vector ρ = (θ, *w*) ∈ *Q* is given by the bias vector θ and the weight matrix *w* of the network *N*(*A*). Thus, one has dim *Q* = *q* = *n* · (*n* + 1).

As a next step we argue that the sensor inputs to the neural system *N*(*A*) can be assumed to act as parameters of the neurodynamics. Because brain-like systems will always act in a sensorimotor loop, the sensor signals *s*(*t*) ∈ *S* will always drive the neurodynamical system *N*(*A*). Assuming in a first approximation that the sensor signals *s*(*t*) change so slowly that the orbits of the neural system are always able to converge to an attractor, then they can be considered as varying parameters. For that reason we will subsume the sensor signals *s*(*t*) as part of the bias terms $\theta \left(t\right):=\hat{\theta }+s\left(t\right)$, with $\hat{\theta }=\text{constant}$.

A neural system then has to be described as a *parametrized family of discrete-time dynamical systems* denoted by (*A, f*; *Q*), with *A* ⊂ ℝ^*n*^ the activation space, *Q* ⊂ ℝ^*q*^ the parameter space, and a differentiable map *f* : *Q* × *A* → *A*. For a specific parameter vector ρ ∈ *Q*, we write *f*_ρ_ : *A* → *A* for the corresponding dynamical system, and denote the *q-parameter family* of neurodynamical systems also by (*A, f*_ρ_), ρ ∈ *Q*. The only varying parameters considered in the following are the bias terms θ_*i*_, *i* = 1, …, *n*. As stated above, other parameters of the animats brain, like synaptic weights *w*_*ij*_ are constant.

**Figure 6:**
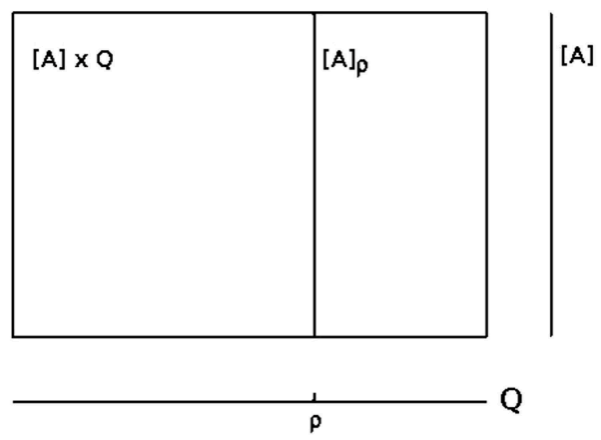
**The fiber structure of a neural system: there is an attractor-landscape [***A***]_**ρ**_ attached to every parameter ρ**.

We may now look at the “brain” as a fiber structure over parameter space *Q* (compare Figure 6): To every ρ ∈ *Q* there is attached the activation space *A* together with the flow ψ_ρ_ corresponding to ρ ∈ *Q*; i.e., there is a whole attractor-landscape, denoted by [*A*]_ρ_, attached to every parameter ρ ∈ *Q*.

### 3.1. Parametric stability

Now, given two different parameter vectors ρ and ρ′ in *Q*, one may ask if the corresponding attractor-landscapes are similar or not in the sense that there exist a homeomorphism carrying oriented orbits onto oriented orbits, especially attractors onto attractors. Using definition 1 we introduce the following

Definition 2. *Given a neurodynamical system* (*A, f*; *Q*)*. Two different parameters* ρ, ρ′ ∈ *Q are said to be* homologous *if the corresponding dynamic systems* (*A, f*_ρ_) *and* $\left(A,f_{\rho^{\prime }}\right)$ *are topologically conjugate; i.e., if the following diagram commutes:*

$$\begin{array}{c} f_{\rho }\\ \psi \;\begin{array}{c} \begin{array}{cc} A & \begin{array}{cc} \to & A \end{array} \end{array}\\ \begin{array}{cc} ↓ & \begin{array}{cc} \; & ↓ \end{array} \end{array}\\ \begin{array}{ccc} A & \to & A \end{array} \end{array}\;\psi \\ f_{\rho^{\prime }} \end{array}$$

**Figure 7:**
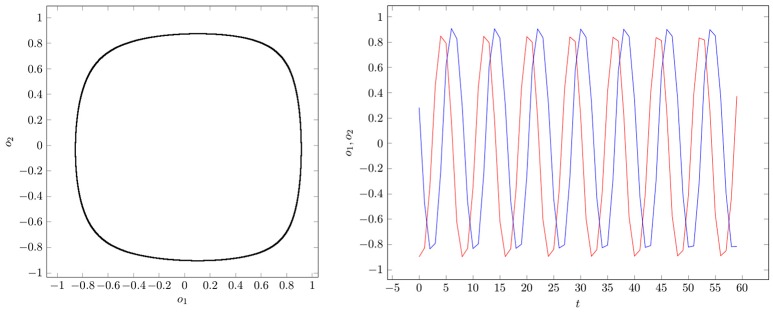
**(Left)** Attractor in (*o*_1_, *o*_2_)-output space. **(Right)** Output signals of the 2-neuron oscillator *Osci*_1_ with parameters given in Table A1.

**Figure 8:**
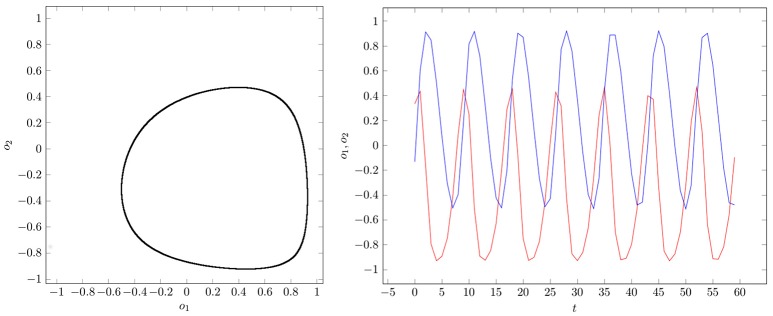
**(Left)** Attractor in (*o*_1_, *o*_2_)-output space. **(Right)** Output signals of the 2-neuron oscillator *Osci*_2_ with parameters given in Table A1.

If two parameter vectors ρ, ρ′ ∈ *Q* are homologous, then the corresponding neurodynamics have qualitative the same behavior; i.e., attractors and basin boundaries may be deformed. In Figures 7, 8, for example, attractors and output signals of an oscillatory 2n-network with two different bias terms are displayed. The two attractor-landscapes [*A*]_ρ_ and ${\left[A\right]}_{\rho^{\prime }}$ corresponding to homologous parameters θ, θ′ are qualitatively, i.e., topologically, the same.

This leads us to an essential concept, that of *parametric stability*, which we define in correspondence to the concept of structural stability in the general theory of dynamical systems (Thom, 1989).

Definition 3. *Given a neurodynamical system* (*A, f*; *Q*) *and a parameter vector* ρ_0_ ∈ *Q*. *Then the system* (*A, f*_ρ_0__) *is called* parametrically stable, *if there exists an* ϵ > 0 *such that for every* ρ ∈ *Q satisfying* ||ρ−ρ_0_|| < ϵ *the systems* (*A, f*_ρ_) *are topologically conjugate to* (*A, f*_ρ_0__).

Definition 4. *Given a neurodynamical system* (*A, f*; *Q*). *The* domain of parametric stability *corresponding to a parameter vector* ρ_0_ ∈ *Q*, *denoted by* $P\left(\rho_{0}\right)\subset Q$*, is the maximally connected parameter set in Q containing all* ρ ∈ *Q which are homologous to* ρ_0_ ∈ *Q*.

Thus, all systems (*A, f*_ρ_) with $\rho \in P\left(\rho_{0}\right)$ are topologically conjugate to (*A, f*_ρ_0__).

Parametrically stable systems are essential for modeling experimental situations: If the experimental inaccuracy is smaller than a domains of parametric stability, then the model remains valid in spite of experimental perturbations. More general, parametric stability is an essential concept, because interesting real (i.e., physical, biological, etc.) phenomena are of course those which are stable under small perturbations of their defining conditions. For instance, a convergent neural network may stay convergent under a small perturbation of their parameters.

### 3.2. Bifurcations

As a second step to describe the dynamics of neural systems we have assumed that the dynamics depends on control parameters, that is, on variables that vary much more slowly than the states of the system. Suppose these parameters change along a smooth path ρ(*t*) ∈ *Q*. If all ρ(*t*) for *t* ∈ [*t*_1_, *t*_2_] are homologous, the corresponding neurodynamical systems will show qualitatively the same behavior, although the attractors and their basins in activation space *A* will move and deform. To such a situation we refer to as a *morphing attractor-landscape* with its *morphing attractors* (Negrello and Pasemann, 2008; Negrello, 2011; Toutounji and Pipa, 2014).

But the path ρ(*t*) may reach a point ρ_*c*_ in parameter space *Q* where the behavior of a system changes qualitatively, i.e., the type and/or numbers of attractors will change, when the path crosses ρ_*c*_. Such points ρ_*c*_ ∈ *Q* are called critical parameters or *bifurcation points*. Thus, bifurcation points are associated with the appearance of topologically non-conjugate systems. The values of ρ_*c*_ ∈ *Q* are called the *bifurcation values*. The appearance of bifurcations in a system are often studied with the help of *bifurcation diagrams*. These are demonstrations of attractor sequences resulting from the variation of only one control parameter (compare **Figure 10**).

The (closed) subspace K⊂Q of all bifurcation points is called the *bifurcation set* of the system (*A, f*; *Q*). Bifurcation sets are sets in *Q* (i.e., curves, surfaces, hyperspaces) which separate different domains of parametric stability.

**Example 2:** As the most simple example we will discuss a single neuron with self-connection *w* as a 2-parameter family of dynamical systems (*A, f*; *Q*) given by

(4) $$\begin{array}{l} a\left(t+1\right)=\theta +w\cdot \tau \left(a\left(t\right)\right),\;t\in \mathbb{Z}, \end{array}$$

(compare also Pasemann, 1993a for a single neuron with logistic function σ(*x*) = (1 + *e^−x^*)^−1^ as transfer function). Stability analysis tells us that for |*w*| < 1 there exist only global fixed points. Otherwise one will find bi-stable systems for *w* > 1, and a domain with global period-2 attractors for *w* < −1. Typical bifurcation diagrams are shown in **Figure 10**.

**Figure 9:**
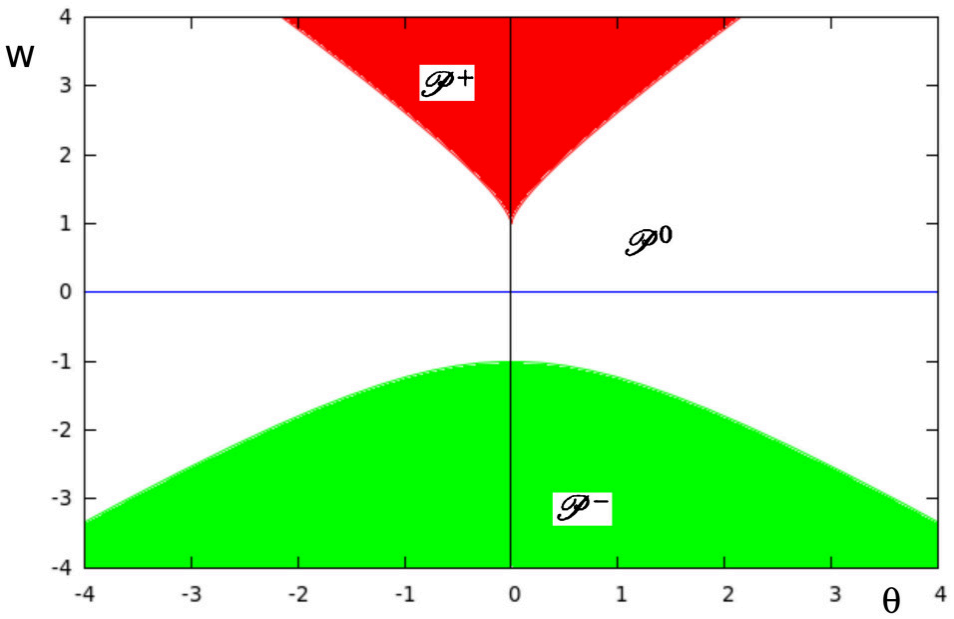
**The parameter space ***Q*** ⊂ ***R***^**2**^ of a single neuron with self-connection ***w*** with its three domains of parametric stability: $P^{0}$ (white) relates to global fixed point attractors, $P^{+}$ (red) to bi-stable systems, and $P^{-}$ (green) to period-2 oscillations**. These domains are separated by bifurcation sets $K^{+}$ and $K^{-}$, respectively.

In Figure 9 the three different domains of parametric stability in *Q* ⊂ *R*^2^ are shown: Here $P^{0}$ (white) denotes the parameter domain for systems having a global fixed point attractor, $P^{+}$ (red) refers to bi-stable systems, and $P^{-}$ (green) to oscillatory systems. They are separated by bifurcation sets $K^{+}$ and $K^{-}$ in *Q*. Thus, a single neuron with self-connection comes in three dynamical forms (compare definition 5).

**Figure 10:**
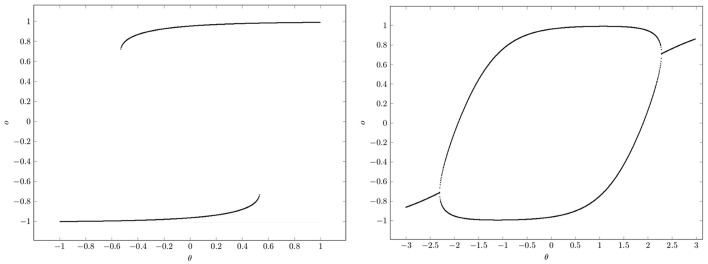
Bifurcation diagrams for output space A^*^ of a single neuron with self-connection ***w***; the bias term θ is varied back and forth over the interval. (Left) Demonstrating bi-stability and hysteresis for *w* = 2.0. **(Right)** Switching on and off a period-2 oscillator for *w* = −2.0.

At $K^{+}$, that is for *w* ≥ 1, there are saddle-node bifurcations, and at $K^{-}$, that is for *w* ≤ −1, there are period-doubling bifurcations. This can be clearly seen in the bifurcation diagrams of Figure 10. They show that a single neuron with positive self-coupling can act as a hysteresis element (short term memory), whereas a neuron with negative self-connection can serve as a switchable oscillator (compare also Pasemann, 1993a).

What should be taken from this simple example is, that in situations where there are parameter domains for which there are coexisting attractors, it depends on the direction from which a path ρ(*t*) in parameter space *Q* hits a bifurcation set K⊂Q (compare Figure 10). This leads to phenomena, called *generalized hysteresis* effects, demonstrating that the development of the system depends crucially on the history of the system. And therefore the behavior of these *path-dependent* systems will not be explicitly deducible from the knowledge of their actual state. This is one reason for the “complexity” of neural systems, and a source of their fascinating faculties.

Having clarified the decisive role of domains of parametric stability P⊂Q for the behavior of parametrized family of dynamical systems, it is natural to associate to a non-critical parameter vector ρ^*^ ∈ *Q* a set of dynamical systems (*A, f*_ρ_) which are parametrically stable with respect to ρ^*^ ∈ *Q*. With reference to the designation of Thom Thom (1989), we give the following

Definition 5. *Given a system* (*A, f*; *Q*)*, and let* ρ_0_ ∈ *Q denote a non-critical parameter vector. A* dynamical form *of* (*A, f*; *Q*) *is a connected set* $F_{\rho_{0}}\subset Diff\left(A\right)$ *of dynamical systems* (*A, f*_ρ_) *which are topologically conjugate to* (*A, f*_ρ_0__).

Assuming that changing parameter values correspond to changing sensor signals, one can deduce that if a sequence of signals stays in a certain domain of parametric stability P, the dynamics of the neural system stays qualitatively the same. And therefore we can assume that the resulting behavior of the controlled system, the animat, will not change dramatically.

## 4. Meta-transients

In the next step we will have to ease the restrictions on the parameters by assuming that the sensor signals can change so fast that the activations *a*(*t*) of the neurodynamical system (*A, f*_ρ_) can not approach an attractor Γ ⊂ *A* asymptotically.

In the following the considered parameters will be the sensor inputs *s*(*t*) of an animat, and all other parameters are fixed. Due to properties of the environment, or due to the behavior of the animat, its sensor inputs may change so fast that they can not be considered as parameters in the strict mathematical sense.

Such a situation is often described in terms of the dynamics of non-autonomous systems. But it is different from the situations covered by control theory (Gardner, 1983; Sussmann, 1983; Respondek, 1996) or by skew-product systems (Kloeden et al., 2013) in so far as a sequence of such sensor inputs is neither the trajectory of a dynamical system in parameter space, nor is it a well defined sequence leading to a preexisting goal. Here the sensor inputs depend on the dynamics of the physical environment (exo-motion) as well as on the movements/actions of the animat itself (ego-motion). We will come to that later again.

Assuming that parameters change almost as fast as the internal states, the resulting sequence of states is no longer that of a transient to one and the same attractor. Suppose the neural system at time *t* is in a state *a*(*t*) on a definite transient *O*(Γ_ρ(*t*)_) to an attractor Γ_ρ(*t*)_ of the neural system (*A, f*_ρ(*t*)_). If the parameter vector a short time later satisfies ρ(*t* + *k*) ≠ ρ(*t*) the corresponding state *a*(*t* + *k*) will be an element of a different transient *O*(Γ_ρ(*t*+*k*)_) to a different attractor Γ_ρ(*t*+*k*)_ ⊂ *A*.

So, let σ_θ_: = {*s*(*t*), *s*(*t* + 1), *s*(*t* + 2), …} denote such a sequence of sensor inputs represented by a sequence of parameter vectors θ(*t*) in *Q*. This will induce a sequence of states α(σ_θ_): = {*a*(*t*), *a*(*t* + 1), *a*(*t* + 2), …} on *A* with

(5) $$\begin{array}{l} a\left(t+1\right)=f_{\rho \left(t\right)}\left(a\left(t\right)\right),\text{ that is},\;a_{i}\left(t+1\right)\\ =\theta_{i}\left(t\right)+\overset{n}{\underset{j=1}{\sum }}w_{ij}a_{j}\left(t\right). \end{array}$$

Such a sequence α(σ_θ_) in *A* will be called a *meta-transient* (Negrello and Pasemann, 2008). Thus, a meta-transient is not a transient of a dynamical system, but it is a sequence of states *a*(*t*) ∈ *A* following the morphing attractors of a sequence of the parametrized dynamical systems (*A, f*_ρ(*t*)_). The projection of such a meta-transient on *A* back to the parameter space *Q* then gives the sequence of “driving” parameter values σ_θ_.

If we define a map Φ:*Q* × *A* → *A* associated with the given parametrized family of dynamical systems by

$$\begin{array}{l} \Phi \left(\rho ,a\right)=f_{\rho }\left(a\right),\;a\in A, \end{array}$$

then the elements of a meta-transient α(σ_θ_) are generated by this map according to

$$\begin{array}{l} \alpha \left(\sigma_{\theta }\right)=\cdots ◦f_{\rho \left(t+2\right)}◦f_{\rho \left(t+1\right)}◦f_{\rho \left(t\right)}\left(a\left(t\right)\right). \end{array}$$

For example, if the input to a neuron with excitatory self-connection is slow when compared with the internal dynamics one will observe a clear hysteresis signal as in Figure 10. If the input signal changes much faster, then there will not be “jumps” at the boundaries of the hysteresis domains but a kind of “squashed” hysteresis loop will appear, as was observed for instance in Manoonpong et al. (2010) for the dynamics resulting from audio input signals.

Furthermore, if all the parameter values, corresponding to the sequence σ_θ_ of sensor inputs, lie in one and the same domain of parametric stability P, the behavior of the animat's body will not change dramatically, and one may describe it as “the same.” But if a sequence of parameter values crosses a bifurcation set K in parameter space *Q* the system may behave in a very different way.

## 5. Projections to motor space *M*

**Figure 11:**
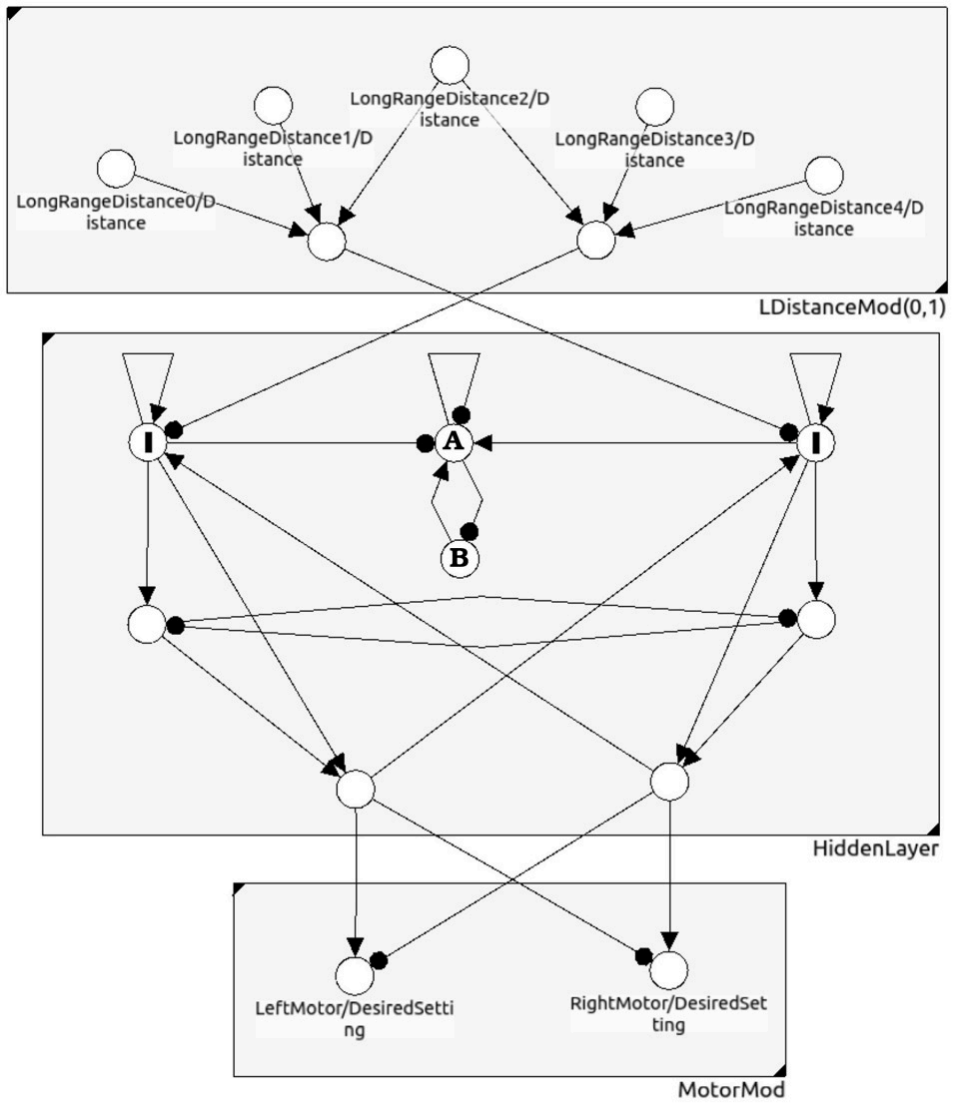
**A neural network for obstacle avoidance behavior of a two-wheeled robot**. One discerns between the sensor space, the so called “brain” (hidden layer), and the motor space.

All the dynamics discussed so far has the goal to generate appropriate body movements. Therefore, the only interesting thing here is the effect of the activities of the neural system which activate the motor neurons. Thus, we have to project the meta-transients α(σ_θ_) on phase space *A* to the motor space *M* with dim (*M*) = *k* < *n*. This projection, denoted by Π : *A* → *M*, is assumed here to correspond to the application of a one-layer feedforward network (compare Figure 11). The activations of the *k* motor neurons then are spanning the output layer, and we define

(6) $$\begin{array}{l} \Pi {\left(a\right)}_{j}:=\overset{n}{\underset{i=1}{\sum }}w_{ji}\tau \left(a_{i}\right),\;a\in A,\;j=1,\ldots ,k, \end{array}$$

where *w*_*ji*_, *i* = 1, …, *n*, *j* = 1, …, *k* denote the weights from the *n* internal neurons to the *k* motor neurons. The activation of the jth motor neuron *m*_*j*_ ∈ *M* having a bias value $\theta_{j}^{M}$ is then given by

(7) $$\begin{array}{l} m_{j}=\theta_{j}^{M}+\Pi {\left(a\right)}_{j},\;a\in A,\;j=1,\ldots ,k. \end{array}$$

Such a motor neuron in general will not be connected to all of the brains neurons. Therefore, there will be many internal states *a* ∈ *A* which will project to identical motor activations *m* ∈ *M*. This will give the second fiber structure of the sensorimotor loop, where the fiber *F*_*m*_ ⊂ *A* over *m* ∈ *M* is given by

(8) $$\begin{array}{l} F_{m}:=\left\{a\in A|\Pi \left(a\right)=m\right\},\;m\in M. \end{array}$$

Then, what is observable is the behavior of the animat generated by a sequence of motor states

(9) $$\begin{array}{l} \mu \left(\sigma_{\theta }\right):=\left\{m\left(t\right),m\left(t+1\right),m\left(t+2\right),\ldots \right\} \end{array}$$

which corresponds to a given meta-transient α(σ_θ_) on *A*; that is, with ρ(*t*) ∈ *Q*, *a*(*t*) ∈ *A*, and bias terms of motor neurons θ^*M*^ ∈ ℝ^*k*^ one has

(10) $$\begin{array}{l} m\left(t\right)=\theta^{M}+\Pi ◦\Phi \left(\rho \left(t\right),a\left(t\right)\right). \end{array}$$

From the projection argument it is clear that not the whole state space *A* is of direct relevance for the behavior of the animat. It is obvious that the activity of neurons not connected to the motor neurons do not have a direct effect on the behavior of the animat. Therefore an attractor in *A*, if it is a fixed point, a periodic orbit or even a chaotic attractor, may be projected to only one and the same motor state *m* ∈ *M*; attractors, their transients or meta-transients may then have little or no effect on motor activities at all.

To reflect this property we introduce a splitting of every state *a* ∈ *A* into a so called *horizontal* and a *vertical* part; i.e.,

(11) $$\begin{array}{l} a=a^{v}+a^{h},\text{ with }\Pi \left(a^{v}\right):=0. \end{array}$$

And due to this splitting we have a direct decomposition of the space of brain states A into horizontal and vertical parts; i.e.,

(12) $$\begin{array}{l} A=A^{v}⊕A^{h}, \end{array}$$

where *A*^*v*^ is given as *A*^*v*^ = ker Π.

Let there be *l* ≥ *k* internal neurons being directly connected with neurons in the motor layer; they serve as an *l*-dimensional input space *B* ⊂ *A*^*h*^ of the feedforward network (compare Figure 11). Furthermore, due to the geometry of feedforward networks (Pasemann, 1993b), in general there is a (*l* − *k*)-dimensional linear subspace *C*_*m*_ ⊂ *B* on which the activation of the motor neurons is constant.

The dynamics directly relevant for behavior then will actually live in the horizontal state space *A*^*h*^ ⊂ *A*. Correspondingly, what will lead to an effective behavior is a sequence of horizontal states given by a *horizontal meta-transient* on *A*

(13) $$\begin{array}{l} \alpha^{h}\left(\sigma_{\theta }\right):=\left\{a^{h}\left(t\right),a^{h}\left(t+1\right),a^{h}\left(t+2\right),a^{h}\left(t+3\right),\ldots \right\}. \end{array}$$

Going back to section 3 let us consider again a discrete-time dynamical systems *f*_ρ_ :*A* → *A* with *fixed* parameter vector ρ ∈ *Q*. Then, post hock, we can introduce a well-defined splitting of the dynamical system *f*_ρ_ into vertical and horizontal parts by

(14) $$\begin{array}{l} f_{\rho }\left(a\right)=f_{\rho }^{v}\left(a^{v}\right)+f_{\rho }^{h}\left(a^{h}\right),\;f_{\rho }^{v}\left(a^{h}\right):=0,\;f_{\rho }^{h}\left(a^{v}\right):=0. \end{array}$$

It is obvious that only the *horizontal dynamics* $f_{\rho }^{h}:A^{h}\to A^{h}$ contributes to the observable behavior of an animat, whereas the *vertical dynamics* $f_{\rho }^{v}:A^{v}\to A^{v}$ will describe brain processes which may be associated to a dynamical kind of memory, to association, planning, dreaming, contemplation, and the like; that is, to the cognitive faculties of the brain.

Furthermore, suppose that two dynamical systems *f*_ρ_ and $f_{\rho \prime }$ with ρ,ρ′∈P, P⊂Q a domain of parametric stability (compare section 3.1), are topologically conjugate. Then it is reasonable that their horizontal components $f_{\rho }^{h}$ and $f_{\rho \prime }^{h}$ will generate motor states in *M* which lead to variants of a specific behavior. The next example gives a demonstration of this situation.

**Example 3:** In evolutionary robotics one often used the motor dynamics of a system as a fitness criterion to reduce the “ineffective” higher dimensional neurodynamics of evolved controllers to analyzable, minimalistic solutions for which the discussed effects could be studied (Wischmann and Pasemann, 2006; von Twickel et al., 2011; Pasemann et al., 2012). Here only a simple example of a neurocontroller may be given by the following recurrent neural network (Figure 11). It provides an obstacle avoiding behavior of a Khepera-like Robot (Toutounji and Pasemann, 2016). It uses five distance sensors (sensor layer) and two motor neurons (motor layer).

The hidden layer (the “brain”) has eight neurons, but only two of them project to the two-dimensional motor space *M*. Though the brain dynamics runs in an 8-dimensional state space *A* only a 2-dimensional subspace *B* ⊂ *A* determines the motor activity directly. What is going on in the 6-dimensional vertical state space has no immediate effect on the behavior of the robot. Indirectly, of course, the dynamics on *A* can influence the behavior of the robot; for instance, the over-critical excitatory self-connections of the input neurons *I* (Figure 11) control the turning angle of the robot at walls. The submodule in *N*(*A*) composed of the two neurons A and B has an interesting dynamics not influencing the motor behavior. They display a “chaotic” meta-transient while the the robot is turning, ending up in a period-2 attractor after a complete right turn, and in a period-4 attractor after a complete left turn. This internal (vertical) dynamics does not contribute to the behavior of the robot, but can be used as a kind of memory for subsequent decisions. The over all performance of this controller is comparable to that of the 2-neuron network called the MRC (minimal recurrent controller) in Hülse et al. (2004).

### 5.1. Closing the loop

**Figure 12:**
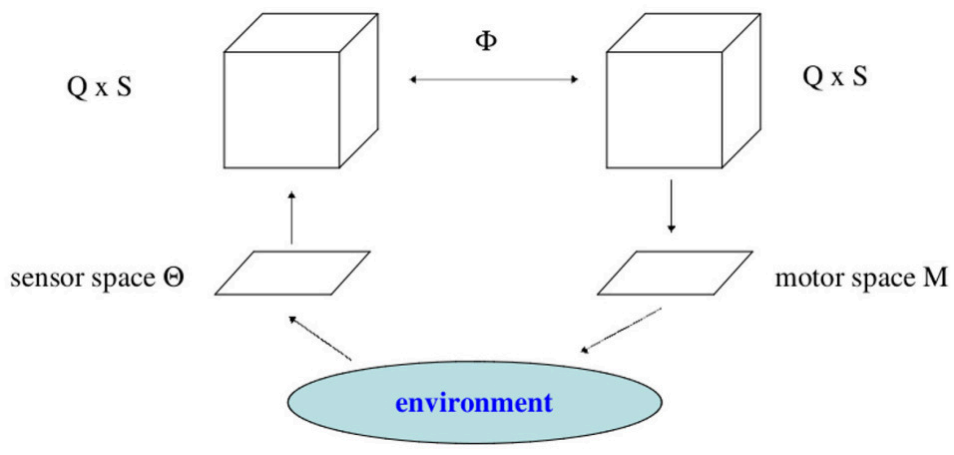
**The sensorimotor loop through the environment**.

Every activity of the motor neurons will change the sensor input to the system (compare Figure 12). In this sense we have a closed loop, and one may call it the *ego-motion-loop*. But the essential point is, that this loop has to go through the environment of the system; i.e., how the motor activity is reflected by the sensor input depends, first, on the appearance and properties of the environment, and second, on processes in the environment itself, called *exo-motion*. This may lead to a discrimination of sensor input variations into those which are due to changes of the motor signals, and those which are due to changes in the environment only (Philipona et al., 2003).

That this inextricable fusion of two influences can not be described as a control theoretical type of closed loop with an additional noise term is clear by two facts: First, what is happening in the physical environment of an animat in general will not be a well defined process, and, second, the motor outputs, as we have seen, are not necessarily a direct reflex of the sensor inputs. Planning, focusing, ignoring performed by the vertical brain activation dynamics are modulating the reaction to sensor inputs. Thus, even formally it is difficult to describe the neural dynamics in the sensorimotor loop in terms of a control-theoretical model.

## 6. Discussion

The description of biological brains as dynamical systems is often assumed to be an appropriate approach to describe cognition and the behavior of animals (Port and Van Gelder, 1995; Thelen and Smith, 1996). Based on the observation that the typical activity of an animat is a reaction to its environment, we used the sensorimotor loop to carefully approach the dynamics hypothesis in three steps. Relying on experiences in the field of evolutionary robotics (Nolfi and Floreano, 2000) we used discrete-time neurodynamics to, first, describe the (isolated) brains as dynamical systems. Having realized that (living) brains are always driven by sensor inputs, we made clear that the description of brains as parametrized families of dynamical systems is more appropriate. This allowed to introduce the concept of parametric stability which helped to formalize the general observation that a certain behavior is robust against “noise,” and can be classified as “the same,” although the initializing sensor inputs vary over a larger domain.

In a third step, assuming that sensor inputs may change so fast that they can not be assumed to serve as parameters in the mathematical sense (compare for instance Manoonpong et al., 2005), we were compelled to introduce the concept of meta-transients to describe the brains activity in a sensorimotor loop. These meta-transients in general will *not* be describable as orbits of a dynamical system. Finally, we used the fact that not all of the brains activity is directly reflected in the motor performance to discern between the brains effective (horizontal) and internal (vertical) activations.

In a more general sense the horizontal part is associated more with the sensorimotor pathways, whereas the vertical part is assigned to the higher centers of the brain, associated with cognitive faculties of a system. Of course horizontal and vertical processes are not decoupled and depend on each other; they are processes on one and the same highly recurrent network. As usual, higher centers are assumed to check the adequacy of the activities along the sensorimotor pathways; they are modulating the sensorimotor flow of signals. On the other hand, the vertical processes are permanently restricted by the “horizontal” flow of signals; otherwise, that is, without sensor inputs, they will run freely into perhaps noxious states of brain and body.

Following a purely formal approach to neurodynamics, we introduced in Section 3.2 the concept of parametric stability and the associated concept of a dynamical form. We think that these concepts may help to discuss questions concerning the representation of objects or, in this context better, behavior relevant situations in the external world.

From the dynamical point of view certain patterns of sensor inputs will be associated with the existence of certain attractors in activation space *A*; or otherwise stated, with the existence of a certain attractor-landscape. Because one has to assume that the brains dynamics is always driven by sensor inputs (including proprioception) it is more plausible to refer to a basin of attraction as a candidate for representing an external situation. Taking our argument for meta-transients serious it becomes obvious, that a dynamical form, associated with a certain type of behavior, is a reasonable representative for behavior relevant situations in the external world. Thus, taking parametric stability as essential for the reproducible identification of “the same” situations gives a reasonable conceptual basis for treating brain dynamics induced by an ever changing complex environment.

If one approves this interpretation then it will also allow for a less restrictive dynamical view on memory. Neural memories usually are represented by asymptotically stable fixed points, like in Hopfield's associative-memory model, or are conceived as periodic, quasiperiodic, or even chaotic attractors of neural networks. In fact, the correspondence between attractors and memories is one of the fundamental aspects of neural networks. But, as we have seen, situated in a sensorimotor loop and driven by sensor inputs, the best we can expect is that attractors of a neural network serve as kinds of symbols, while the system always runs on transients to these attractors (or on meta-transients). So in a first step memory should be associated with the basins of certain attractors. Taken that the natural situation is such that neural systems in the sensorimotor loop run on meta-transients, we have to assume that the union of all basins of attraction, belonging to the possibly morphing attractors of a dynamic form, should be identified with the memory of certain behavior relevant external situations. We will call this kind of memory model a *blurred* memory. The relation between learning and blurred memory will be the subject of further research.

## 7. Author contributions

The author confirms being the sole contributor of this work and approved it for publication.

### Conflict of interest statement

The author declares that the research was conducted in the absence of any commercial or financial relationships that could be construed as a potential conflict of interest.

## Appendix

The following is a list of parameters corresponding to the designated 2-neuron networks used for demonstrations in this paper.

**Table A1:** **Parameters for 2-neuron neural networks (Equation 2) discussed in the paper**.

**System**	**θ_1_**	**θ_2_**	***w*_11_**	***w*_12_**	***w*_21_**	***w*_22_**
sys1	−2.0	0.0	0.0	1.6	−1.6	0.0
sys2	−1.6	0.0	−3.0	1.6	−1.6	0.0
sys3	0.0	0.0	1.1	0.6	−0.6	0.9
sys4	−3.0	0.0	−4.8.	2.3	−2.3	0.0
sys5	0.0	0.0	−1.5	1.5	1.5	1.5
sys6	−2.51	0.0	−2.75	1.5	−1.5	0.0
Osci_1_	0.0	0.1	1.0	−1.0	1.0	1.0
Osci_2_	0.0	0.5	1.0	−1.0	1.0	1.0

## References

[B1] Abraham R. Shaw C. D. (1992). Dynamics: The Geometry of Behavior, Vol. 2 Redwood City, CA: Addison-Wesley.

[B2] Ashby W. (1960). Design for a Brain: The Origin of Adaptive Behaviour. London: Chapman & Hall.

[B3] Beer R. D. (1995). A dynamical systems perspective on agent-environment interaction. Artif. Intell. 72, 173–215. 10.1016/0004-3702(94)00005-L

[B4] Bishop G. (1960). Feedback through the environment as an analog of brain functioning, in Self-Organizing Systems, eds Yovitts M. C. Cameron S. (New York, NY: Pergamon), 122–152.

[B5] Dean J. (1998). Animats and what they can tell us. Trends Cogn. Sci. 2, 60–67. 10.1016/S1364-6613(98)01120-6 21227067

[B6] Di Paolo E. A. (2003). Organismically-inspired robotics: homeostatic adaptation and teleology beyond the closed sensorimotor loop, in Dynamical Systems Approach to Embodiment and Sociality, eds Murase K. Asakura T. (Adelaide: Advanced Knowledge International), 19–42.

[B7] Gardner R. B. (1983). Differential Geometric Methods Interfacing Control Theory. Boston, MA: Birkhauser.

[B8] Guillot A. Meyer J.-A. (2001). The animat contribution to cognitive systems research. Cogn. Sys. Res. 2, 157–165. 10.1016/S1389-0417(01)00019-5

[B9] Hirsch M. W. Smale S. Devaney R. L. (2012). Differential Equations, Dynamical Systems, and an Introduction to Chaos. Oxford: Academic Press.

[B10] Hülse M. Wischmann S. Manoonpong P. von Twickel A. Pasemann F. (2007). Dynamical systems in the sensorimotor loop: On the interrelation between internal and external mechanisms of evolved robot behavior, in 50 Years of Artificial Intelligence, eds Lungarella M. Iida F. Bongard J. Pfeifer R. (Berlin: Springer), 186–195.

[B11] Hülse M. Wischmann S. Pasemann F. (2004). Structure and function of evolved neuro-controllers for autonomous robots. Connect. Sci. 16, 249–266. 10.1080/09540090412331314795

[B12] Kloeden P. Pötzsche C. Rasmussen M. (2013). Discrete-time nonautonomous dynamical systems, in Stability and Bifurcation Theory for Non-Autonomous Differential Equations, eds Johnson R. Pera M. P. (Berlin: Springer), 35–102.

[B13] Manoonpong P. Pasemann F. Fischer J. Roth H. (2005). Neural processing of auditory signals and modular neural control for sound tropism of walking machines. Int. J. Adv. Rob. Sys. 2, 223–234. 10.5772/5786

[B14] Manoonpong P. Pasemann F. Kolodziejski C. Wörgötter F. (2010). Designing simple nonlinear filters using hysteresis of single recurrent neurons for acoustic signal recognition in robots, in International Conference on Artificial Neural Networks (Berlin: Springer), 74–383.

[B15] Marcus C. Westervelt R. (1989). Dynamics of iterated-map neural networks. Phys. Rev. A 40:501. 10.1103/physreva.40.501 9901925

[B16] Negrello M. (2011). Invariants of Behavior: Constancy and Variability in Neural Systems. New York, NY: Springer Science and Business Media.

[B17] Negrello M. Pasemann F. (2008). Attractor landscapes and active tracking: the neurodynamics of embodied action. Adaptive Behav. 16, 196–216. 10.1177/1059712308090200

[B18] Nolfi S. Floreano D. (2000). Evolutionary Robotics: The Biology, Intelligence, and Technology of Self-organizing Machines. Cambridge: MIT Press.

[B19] Pasemann F. (1993a). Dynamics of a single model neuron. Int. J. Bifurcat. Chaos 2, 271–278.

[B20] Pasemann F. (1993b). Geometry of feedforward networks, in Classical and Quantum Systems - Foundations and Symmetries, eds Doebner H. D. Scherer W. Schroeck F. (Singapore: World Scientific), 784–790.

[B21] Pasemann F. (2002). Complex dynamics and the structure of small neural networks. Network 13, 195–216. 10.1080/net.13.2.195.216 12061420

[B22] Pasemann F. Rempis C. W. von Twickel A. (2012). Evolving humanoid behaviors for language games, in Language Grounding in Robots, eds Steels L. Hild M. (New York, NY: Springer), 67–86.

[B23] Philipona D. O'regan J. Nadal J.-P. Coenen O. (2004). Perception of the structure of the physical world using unknown sensors and effectors. Adv. Neural Inf. Process. Syst. 16, 945–952.

[B24] Philipona D. O'Regan J. K. Nadal J.-P. (2003). Is there something out there? inferring space from sensorimotor dependencies. Neural computat. 15, 2029–2049. 10.1162/089976603322297278 12959664

[B25] Port R. F. Van Gelder T. (1995). Mind as Motion: Explorations in the Dynamics of Cognition. Cambridge: MIT Press.

[B26] Respondek W. (1996). Geometric methods in nonlinear control theory, in Neural Adaptive Control Technology, eds Zbikowski R. Hunt K. (London: World Scientific), 115–152.

[B27] Sándor B. Jahn T. Martin L. Gros C. (2015). The sensorimotor loop as a dynamical system: how regular motion primitives may emerge from self-organized limit cycles. arXiv preprint arXiv:1511.04338.

[B28] Strogatz S. H. (2014). Nonlinear Dynamics and Chaos: With Applications to Physics, Biology, Chemistry, and Engineering. Boulder, CO: Westview Press.

[B29] Sussmann H. J. (1983). Lie brackets, real analyticity and geometric control. Differ. Geomet. Control Theory 27, 1–116.

[B30] Thelen E. Smith L. B. (1996). A Dynamic Systems Approach to the Development of Cognition and Action. Cambridge: MIT Press.

[B31] Thom R. (1989). Structural Stability and Morphogenesis. Boston, MA: Addison Wesley Publishing Company.

[B32] Toutounji H. Pasemann F. (2016). Autonomous learning needs a second environmental feedback loop, in Computational Intelligence, eds Merelo J. J. Rosa A. Cadenas J. M. Correia A. D. Madani K. Ruano A. Filipe J. (Berlin: Springer), 455–472.

[B33] Toutounji H. Pipa G. (2014). Spatiotemporal computations of an excitable and plastic brain: neuronal plasticity leads to noise-robust and noise-constructive computations. PLoS Comput. Biol. 10:e1003512. 10.1371/journal.pcbi.1003512 24651447 PMC3961183

[B34] Von Foerster H. (1960). On self-organizing systems and their environments, in Self-Organizing Systems, eds Yovitts M. C. Cameron S. (New York, NY: Pergamon), 31–50.

[B35] von Twickel A. Büschges A. Pasemann F. (2011). Deriving neural network controllers from neuro-biological data: implementation of a single-leg stick insect controller. Biol. Cybern. 104, 95–119. 10.1007/s00422-011-0422-1 21327828

[B36] Wang X. (1991). Period-doublings to chaos in a simple neural network: An analytical proof. Complex Sys. 5, 425–44.

[B37] Wilson H. R. Cowan J. D. (1972). Excitatory and inhibitory interactions in localized populations of model neurons. Biophys. J. 12, 1. 4332108 10.1016/S0006-3495(72)86068-5 PMC1484078

[B38] Wischmann S. Pasemann F. (2006). The emergence of communication by evolving dynamical systems, in International Conference on Simulation of Adaptive Behavior (Berlin: Springer), 777–788.

[B39] Zahedi K. Ay N. Der R. (2010). Higher coordination with less controla result of information maximization in the sensorimotor loop. Adapt. Behav. 18, 338–355. 10.1177/1059712310375314

